# Differential Influence of Inositol Hexaphosphate on the Expression of Genes Encoding TGF-****β**** Isoforms and Their Receptors in Intestinal Epithelial Cells Stimulated with Proinflammatory Agents 

**DOI:** 10.1155/2013/436894

**Published:** 2013-12-29

**Authors:** Małgorzata Kapral, Joanna Wawszczyk, Stanisław Sośnicki, Ludmiła Węglarz

**Affiliations:** Department of Biochemistry, Medical University of Silesia, Jedności 8, 41-200 Sosnowiec, Poland

## Abstract

Transforming growth factor **β** (TGF-**β**) is a multifunctional cytokine recognized as an important regulator of inflammatory responses. The effect of inositol hexaphosphate (IP6), a naturally occurring phytochemical, on the mRNA expression of TGF-**β**1, TGF-**β**2, TGF-**β**3 and T**β**RI, T**β**RII, and T**β**RIII receptors stimulated with bacterial lipopolysaccharides (*Escherichia coli* and *Salmonella typhimurium*) and IL-1**β** in intestinal cells Caco-2 for 3 and 12 h was investigated. Real-time qRT-PCR was used to validate mRNAs level of examined genes. Bacterial endotoxin promoted differential expression of TGF-**β**s and their receptors in a time-dependent manner. IL-1**β** upregulated mRNA levels of all TGF-**β**s and receptors at both 3 h and 12 h. IP6 elicited the opposed to LPS effect by increasing downregulated transcription of the examined genes and suppressing the expression of TGF-**β**1 at 12 h. IP6 counteracted the stimulatory effect of IL-1**β** on TGF-**β**1 and receptors expression by decreasing their mRNA levels. IP6 enhanced LPS- and IL-1**β**-stimulated mRNA expression of TGF-**β**2 and -**β**3. Based on these studies it may be concluded that IP6 present in the intestinal milieu may exert immunoregulatory effects and chemopreventive activity on colonic epithelium under inflammatory conditions or during microbe-induced infection/inflammation by modulating the expression of genes encoding TGF-**β**s and their receptors at transcriptional level.

## 1. Introduction

Inflammatory bowel disease (IBD) is a chronic inflammation of the gastrointestinal tract thought to be a result of dysregulated or aberrant immune response to intestinal flora and multiple environmental factors with regard to genetic predisposition [1]. Exposure of intestinal epithelial cells (IEC) to bacterial components and products can potentially initiate intestinal inflammation by their release of cytokines chemokines and recruitment of inflammatory cells. IEC can also respond to a broad array of cytokines with altered gene expression and growth characteristics [2]. Cytokine that plays a crucial role in the inflammatory diseases such as IBD is IL-1*β*. Enhanced level of this cytokine has been determined in mucosal tissues infected with enteropathogenic bacteria, as well as in mucosal biopsies with active IBD. IL-1*β* activates intracellular signaling cascades in IEC leading to the increase of expression and secretion of proinflammatory cytokines and chemokines, uncontrolled intestinal inflammation, and disruption of epithelial function [3, 4]. Lipopolysaccharide (LPS) or endotoxin, the key component of the cell wall of Gram-negative bacteria, stimulates activation of transcription factors and production of proinflammatory cytokines. The expression of LPS specific Toll-like receptor 4 (TLR-4) in human colorectal cancer cells highlighted a key function of TLR system in the development of colitis-associated tumors, suggesting a role of this receptor in colorectal cancer development and progression [2]. Cytokines with anti-inflammatory properties have been implicated in the prevention of inappropriate immune activation by intestinal flora.

Transforming growth factor *β* is a strong anti-inflammatory cytokine with multipotent mechanism of action [5]. In mammals three isoforms (TGF-*β*1, -*β*2, and -*β*3) have been described, which share 75% amino acid sequence homology but are encoded by different genes [6]. TGF-*β*s have strong impact on the inflammatory responses and tumor microenvironment including fibroblasts, endothelial cells and immune cells. TGF-*β*s suppress cytotoxic T-cell differentiation and inhibit NK cell and neutrophil effector functions. Also, they have been shown to suppress MHC I and MHC II expression [7]. On the other hand, overexpression of TGF-*β* could induce the secretion of proinflammatory cytokines, for example, TNF-*α* [8], IL-1*β*, IL-6, and IL-8 [7]. This indicates complexity of TGF-*β* signalling immune regulation within different contexts [9]. TGF-*β* signalling is mediated by three specific types of cell surface proteins: TGF-*β* receptor I (T*β*RI), II (T*β*RII), and III (T*β*RIII). T*β*RIII is a coreceptor modulating intracellular TGF-*β*s activities [10]. TGF-*β* initiates its signaling by binding to T*β*RII, which has intrinsic serine-threonine kinase activity. Then, T*β*RII recruits and phosphorylates T*β*RI, establishing heterotetrameric complex consisting of two T*β*RII and two T*β*RI [11]. T*β*RI initiates phosphorylation of the adaptor proteins SMAD2 and SMAD3 which is followed by the formation of complex with SMAD4 [5]. Nuclear SMAD complexes bind to SMAD-binding elements on DNA, affecting transcriptional activity which is dependent on their interaction with coactivators [12]. SMAD7 differs structurally from other members of SMAD family and functions as a negative regulator of TGF-*β* signaling. Its gene expression is induced by TGF-*β*; thus SMAD7 represents negative feedback loop, restraining TGF-*β* activity [13]. It recruits the GADD34 complex to the T*β*RI, thus preventing SMAD2/SMAD3 phosphorylation and TGF-*β* signal transduction. SMAD7 also contributes to T*β*RI dephosphorylation and ubiquitination and proteasomal degradation of the TGF-*β* receptor complex [14]. Many factors modulate the TGF-*β*-mediated cellular response. Transcription factors, histone readers, modifiers, and chromatin remodelers that bind to activated SMAD determine what genes and how they will be affected by signal transduction complexes. TGF-*β* can also activate other non-SMAD signalling pathways, including PI3K, MAPK, TRAF6, and mTORC [15]. Some of the important downstream targets of TGF-*β* signaling include cell cycle checkpoint genes, the activation of which leads to growth arrest. Yet, TGF-*β* signaling can also directly stimulate the production of several mitogenic growth factors which can drive the carcinogenic process [16]. A number of inflammatory diseases including inflammatory bowel disease and cancer are associated with abnormal TGF-*β*s regulation [5, 17, 18]. Monteleone et al. [19] have shown marked overexpression of SMAD7 in the inflamed tissue of IBD patients. Moreover it was associated with a reduction in SMAD3 phosphorylation, which is crucial for anti-inflammatory action of TGF-*β*. Proinflammatory stimuli, such as TNF-*α*, IL-1*β*, and INF-*γ*, also induce SMAD7 expression [20]. Chronic inflammation has been recognized to be associated with a high cancer risk and may be involved in all stages of tumor development, that is, initiation, promotion, and progression [21]. The control of colitis by certain anti-inflammatory agents reduced colon cancer incidence [22].

Colorectal cancer is one of the most common cancers, accounting for 8% of all cancer deaths, making it the fourth cause of cancer deaths[23]. Due to high mortality and extensive anticancer drugs toxicity there has been growing interest in substances that may have chemopreventive action, that is, can prevent or delay the development of cancer [24, 25]. Diet has been proved to play a significant role in the aetiology of colorectal cancer. Consumption of dietary components with anti-inflammatory activity has been associated with reduced risk of developing colorectal cancer. One of the essential components of high fiber diet is inositol hexaphosphate (IP6) [26]. It is a naturally occurring hexaphosphorylated carbohydrate, found in both plant and mammalian cells [27]. With intracellular concentration of about 100 *μ*M, IP6 participates in a variety of cellular functions such as signal transduction, regulation of cell proliferation, and differentiation [28]. IP6 has been shown in *in vitro* studies to inhibit growth of human breast, colon, prostate, and liver cancer cells. Its anticancer properties have been documented to result from its antiproliferative, proapoptotic, and antiangiogenic effects. IP6 is also known for its antioxidant properties, prevention against formation of kidney stones, high blood cholesterol level and heart and liver diseases [27]. Its antioxidant action was recognized in experimental models of myocardial reperfusion injury, pulmonary inflammation, and peptic ulcer induction [28]. Therefore, IP6 is believed to have potential to serve as preventive agent for chronic inflammation and carcinogenesis [29]. Recently, it has been revealed that IP6 has strong impact on transcriptional activity of TGF-*β*s and their receptor genes in colon cancer cells [30].

The aim of the present study was to examine the potential of IP6 to affect proinflammatory agents-influenced changes in transcriptional activity of the genes encoding TGF-*β*1, TGF-*β*2, and TGF-*β*3 and their receptors T*β*RI, T*β*RII, and T*β*RIII in human colon Caco-2 cells.

## 2. Materials and Methods

### 2.1. Cell Culture and Cell Stimulation Assays

The Caco-2 human intestinal epithelial cells (DSMZ, Braunschweig, Germany) were routinely cultured in RPMI 1640 medium (Sigma Aldrich) supplemented with 10% fetal bovine serum (GibcoBRL), 100 U/mL penicillin and 100 *μ*g/mL streptomycin (both from Sigma Aldrich) and 10 mM HEPES (GibcoBRL). They were maintained at 37°C in a 5% CO_2_ atmosphere within a humidified incubator. Cells were seeded into six-well plates (Nunc International) at a density of 4.5 × 10^5^ per well and allowed to grow to 80% confluency in 3 mL of medium. After three days the culture media were changed to media with 2% FBS and cells were then cultured for 2 days. They were then stimulated with 100 *μ*g/mL LPS (*Escherichia coli* serotype 055:B5, *Salmonella enterica* serotype typhimurium; both from Sigma Aldrich), or 1 ng/mL IL-1*β* (Sigma Aldrich) for 30 min. Afterwards cells were treated with 2.5 mM IP6 as dipotassium salt (distilled water dissolved and pH 7.4 adjusted) (Sigma Aldrich) for 3 and 12 h. In separate cultures, cells were incubated with LPS or IL-1*β* at the indicated concentrations and for the indicated times. The untreated Caco-2 cells were used as the control.

### 2.2. RNA Extraction

Total RNA was extracted from cells using TRIzol reagent (Invitrogen) according to the manufacturer's specifications. Integrity of the RNA extracts was qualitatively checked by electrophoresis in 1.0% agarose gel stained with ethidium bromide. RNA concentration was determined spectrophotometrically on the basis of absorbance values at a wavelength of 260 nm using a GeneQuant pro (Amersham Biosciences).

### 2.3. Real-Time qRT-PCR Assay

Detection of the expression of genes encoding TGF-*β* isoforms and their receptors was carried out using a qRT-PCR technique with a SYBR Green chemistry (SYBR Green Quantitect RT-PCR Kit, Qiagen) and Opticon DNA Engine Continuous Fluorescence detector (MJ Research) as described previously [31]. Oligonucleotide primers specific for TGF-*β*1, TGF-*β*2, TGF-*β*3, T*β*RI, T*β*RII, and T*β*RIII mRNAs were designed using Primer Express 2.0 software (PE Applied Biosystems, USA) (Table 1). The thermal profile for one-step RT-PCR was as follows: 50°C for 30 min for reverse transcription and 95°C for 15 min followed by 45 cycles at 94°C for 15 s, 55°C for 30 s, and 72°C for 45 s for amplification. Following RT-PCR, the samples were subjected to temperature ramp from 60°C to 95°C at the rate of 0.2°C/s with continuous fluorescence monitoring for melting curve analysis. Each gene analysis was performed in triplicate. A commercially available standard of *β*-actin (TaqMan DNA Template Reagent Kit, Applied Biosystems) was used to estimate the mRNA copy numbers of examined genes. The obtained results of mRNA copy number were recalculated per *μ*g of total RNA. The expression level of examined genes in cultured cells was expressed as the fold change relative to the control. The value of fold change >1 reflects increased expression of the target gene, and a value of fold change <1 points to a decrease in the gene expression. Finally, specificity of RT-PCR reaction was confirmed by determining the characteristic temperature of melting for each amplimer and by 6% polyacrylamide gel (PAA) electrophoresis of RT-PCR products with their visualization using silver staining.

### 2.4. Statistical Analysis

The results were collected based on three independent experiments. Statistical analysis was performed with the use of Statistica PL 9.0 software. All the results are expressed as means ± SD. Comparison of two data sets was performed by unpaired *t*-test. Significance level was assumed for *P* < 0.05.

## 3. Results

The colon cancer cells Caco-2 showed constitutive expression of genes encoding all three TGF-*β* isoforms and their receptors.

### 3.1. Changes of TGF-*β*1 Expression by the Effect of Proinflammatory Agents and IP6

**Figure 1:**
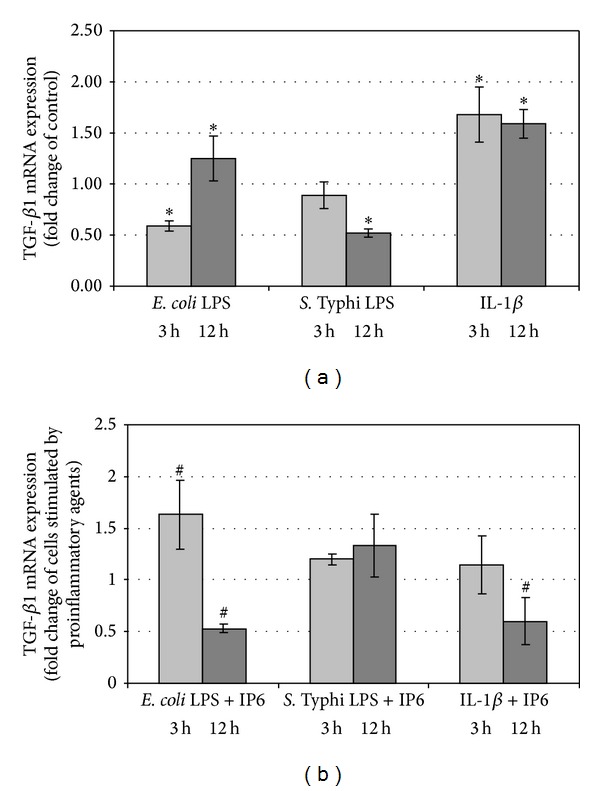
Expression of TGF-*β*1 gene in Caco-2 cells as determined by real-time RT-PCR. Changes in TGF-*β*1 mRNA expression in Caco-2 cells after treatment with (a) proinflammatory agents and (b) proinflammatory agents and 2.5 mM IP6 for 3 h and 12 h. The results are presented as mean ± SD of three separate experiments; *P < 0.05 versus control Caco-2 cells; ^#^ *P* < 0.05 versus proinflammatory agents-stimulated cells.

In the time course of the experiment, differential TGF-*β*1 expression after exposure of Caco-2 to *E. coli* LPS was observed. At 3 h, it decreased in comparison to control (*P* = 0.025) and IP6 up regulated LPS-evoked effect (*P* = 0.032). A significantly higher TGF-*β*1 mRNA level was determined following cell treatment with LPS for 12 h than in unstimulated cells (*P* = 0.047). LPS-stimulated transcription of this gene was remarkably down-regulated by IP6 at that time (*P* = 0.01) (Figures 1(a) and 1(b)). Endotoxin of *S. typhimurium* had no influence on TGF-*β*1 mRNA level after 3 h treatment (*P* = 0.487) but longer exposure of cells to it (12 h) caused significant decrease in transcription of the gene (*P* < 0.001) (Figure 1(a)). The levels of TGF-*β*1 mRNA in cells stimulated with *S*. Typhi. LPS and cells treated with both LPS and IP6 revealed no statistically significant differences after 3 and 12 h (*P* > 0.05) (Figure 1(b)). Incubation of Caco-2 with IL-1*β* for both 3 and 12 h up-regulated TGF-*β*1 gene as compared with untreated cells (*P* < 0.05) (Figure 1(a)). After 3 h, 2.5 mM IP6 did not change TGF-*β*1 expression stimulated by IL-1*β* (*P* = 0.268). Furthermore, significant decrease in the expression of this gene was revealed in cells exposed to IL-1*β* and IP6 for 12 h (*P* = 0.047) in comparison to the cultures treated with IL-1*β* only (Figure 1(b)).

### 3.2. Changes of TGF-*β*2 Expression by the Effect of Proinflammatory Agents and IP6

**Figure 2:**
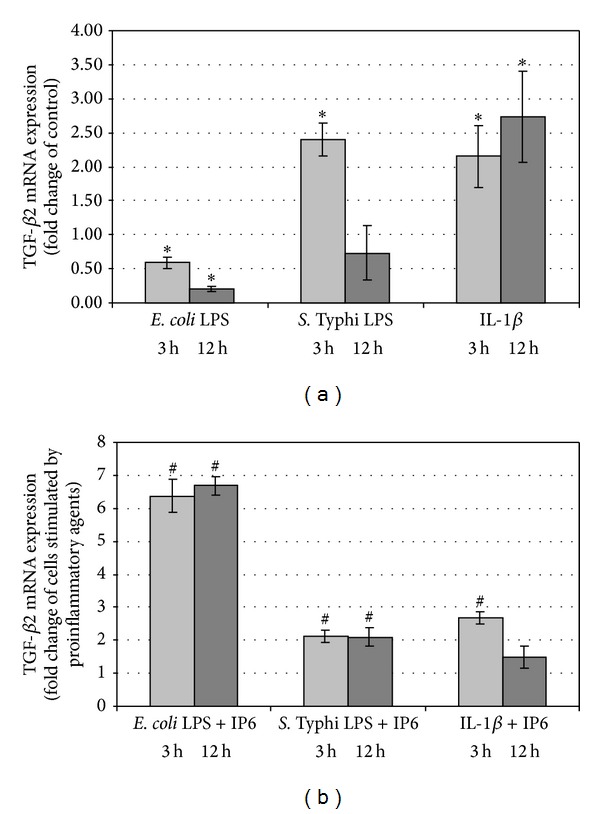
Expression of TGF-*β*2 gene in Caco-2 cells as determined by real-time RT-PCR. Changes in TGF-*β*2 mRNA expression in Caco-2 cells after treatment with (a) proinflammatory agents and (b) proinflammatory agents and 2.5 mM IP6 for 3 h and 12 h. The results are presented as mean ± SD of three separate experiments; *P < 0.05 versus Caco-2 cells; ^#^ *P* < 0.05 versus proinflammatory agents-stimulated cells.

LPS of *E. coli* gradually down-regulated TGF-*β*2 expression within 3–12 h (*P* < 0.05) (Figure 2(a)) and IP6 was able to enhance it markedly at both time points in comparison to LPS effects only (*P* < 0.05) (Figure 2(b)). In response to LPS of *Salmonella* Caco-2 exhibited significantly higher transcription of this gene than control after 3 h (*P* = 0.001). However, the prolongation of time to 12 h led to insignificantly reduced TGF-*β*2 expression (*P* = 0.130) (Figure 2(a)). IP6 enhanced LPS-stimulated transcription of this gene after 3 h (*P* < 0.001). Subsequently (12 h), the combination of IP6 and LPS gave rise to 2-fold increase in TGF-*β*2 mRNA level (*P* = 0.002) compared to LPS-treated cells (Figure 2(b)). The TGF-*β*2 transcript was over 2-fold higher by the treatment with IL-1*β* for 3 h (*P* = 0.007) and 12 h (*P* = 0.001) as compared to control (Figure 2(a)). When IP6 was added to IL-1*β*-prestimulated cultures, the level of TGF-*β*2 mRNA markedly raised at 3 h in comparison to those treated with IL-1*β* alone (*P* < 0.0001) (Figure 2(b)).

### 3.3. Changes of TGF-*β*3 Expression by the Effect of Proinflammatory Agents and IP6

**Figure 3:**
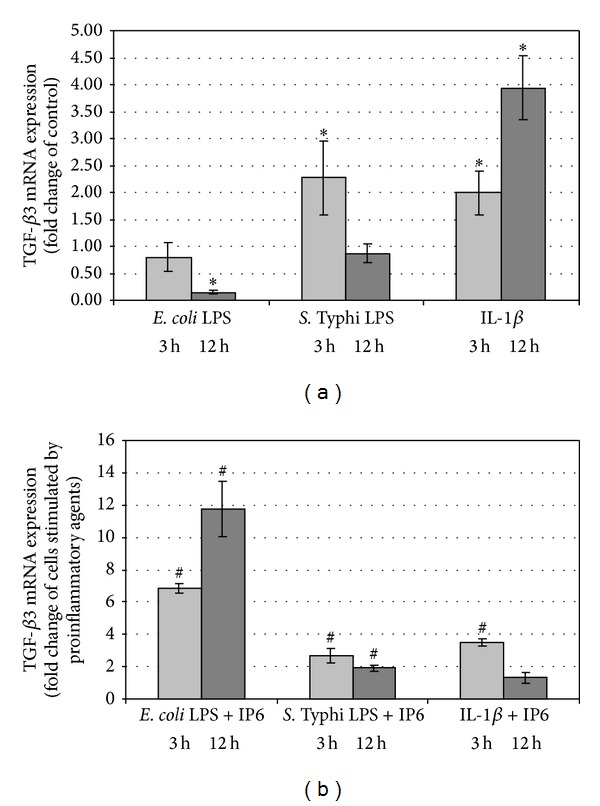
Expression of TGF-*β*3 gene in Caco-2 cells as determined by real-time RT-PCR. Changes in TGF-*β*3 mRNA expression in Caco-2 cells after treatment with (a) proinflammatory agents and (b) proinflammatory agents and 2.5 mM IP6 for 3 h and 12 h. The results are presented as mean ± SD of three separate experiments; *P < 0.05 versus control Caco-2 cells; ^#^ *P* < 0.05 versus proinflammatory agents-stimulated cells.

*E. coli* LPS diminished transcriptional activity of TGF-*β*3 gene in Caco-2 cells in a time-dependent manner. The decrease of the TGF-*β*3 mRNA level was statistically significant compared to control at 12 h (*P* < 0.0001) (Figure 3(a)). IP6 enhanced the expression of this gene after 3 h (*P* < 0.0001) and 12 h (*P* < 0.0001) with reference to LPS-stimulated cells (Figure 3(b)). Cell cultures treated with LPS of *S*. Typhi manifested above 2-fold increase in TGF-*β*3 mRNA expression compared to control culture at 3 h (*P* = 0.016) and IP6 markedly enhanced LPS-induced expression of this isoform (*P* = 0.008). Incubation of cells with *S*. Typhi LPS for 12 h did not change mRNA level of TGF-*β*3 compared to control (*P* = 0.286). In cells exposed to LPS and IP6 statistically significant increase in mRNA for TGF-*β*3 in relation to cells treated with *S*. Typhi LPS only (*P* = 0.005) was observed (Figures 3(a) and 3(b)). IL-1*β* induced transcriptional activity of this gene by 2-fold after 3 h (*P* = 0.009) and about 4-fold after 12 h (*P* < 0.0001) as compared to unstimulated cells (Figure 3(a)). IP6 modified IL-1*β* effects by significant increasing of TGF-*β*3 mRNA expression at 3 h (*P* < 0.001) (Figure 3(b)).

### 3.4. The Effect of IP6 on T*β*RI Expression in Caco-2 Cells Stimulated by Bacterial Endotoxins and IL-1*β*

**Figure 4:**
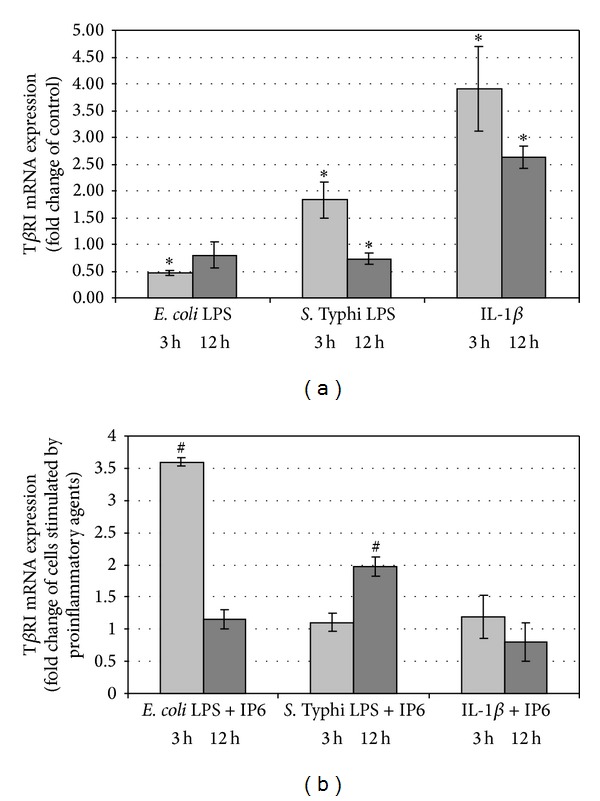
Expression of T*β*RI gene in Caco-2 cells as determined by real-time RT-PCR. Changes in T*β*RI mRNA expression in Caco-2 cells after treatment with (a) proinflammatory agents and (b) proinflammatory agents and 2.5 mM IP6 for 3 h and 12 h. The results are presented as mean ± SD of three separate experiments; *P < 0.05 versus control Caco-2 cells; ^#^ *P* < 0.05 versus proinflammatory agents-stimulated cells.

Lipopolysaccharide of *E. coli* downregulated transcriptional activity of the gene encoding type I TGF-*β* receptor in Caco-2 at 3 h (*P* < 0.001) (Figure 4(a)) while IP6 strongly induced it (*P* < 0.0001) (Figure 4(b)). At 12 h, the expression of the gene in control and LPS-stimulated cultures was comparable (*P* = 0.075), and there were no changes in T*β*RI mRNA amount in the cells treated with LPS and IP6 (*P* = 0.479) (Figures 4(a) and 4(b)). Caco-2 exposed to LPS of *Salmonella* Typhi for 3 h produced significantly higher quantity of T*β*RI transcript than control (*P* < 0.001). The amount of T*β*RI mRNA in cells treated with LPS/IP6 and LPS-stimulated cells was similar (*P* > 0.05). Treatment of Caco-2 cells with *Salmonella* Typhi LPS for 12 h resulted in statistically significant decrease in T*β*RI gene transcription (*P* = 0.016) which was remarkably up-regulated by IP6 (*P* < 0.004) (Figures 4(a) and 4(b)). By comparison, IL-1*β* induced transcriptional activity of T*β*RI gene in Caco-2 cells. The extent of stimulation by this cytokine was 3.9- and 2.6-fold after 3 h and 12 h, respectively, compared to the control (*P* < 0.05) (Figure 4(a)). Nevertheless, 2.5 mM IP6 did not significantly change mRNA T*β*RI expression in IL-1*β*-treated cultures throughout the time period of the experiment (*P* > 0.05) (Figure 4(b)).

### 3.5. The Effect of IP6 on T*β*RII Expression in Caco-2 Cells Stimulated by Bacterial Endotoxins and IL-1*β*

**Figure 5:**
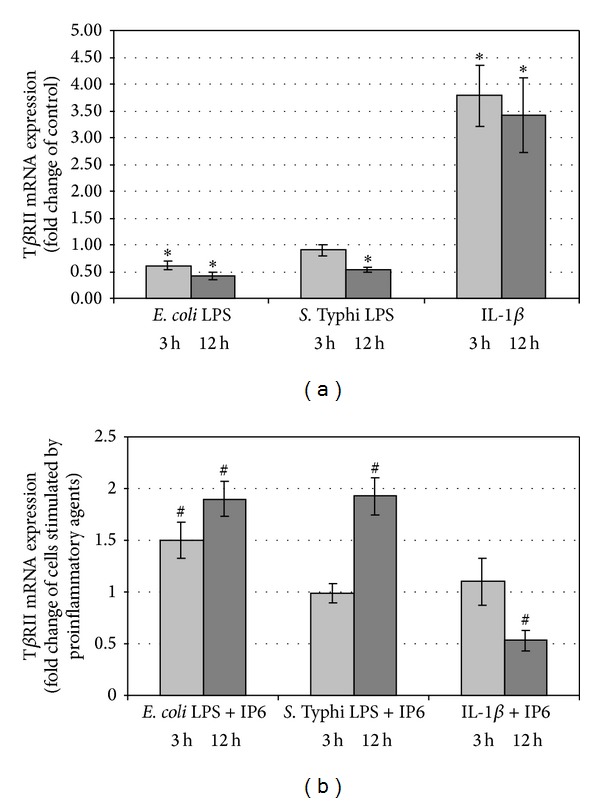
Expression of T*β*RII gene in Caco-2 cells as determined by real-time RT-PCR. Changes in T*β*RII mRNA expression in Caco-2 cells after treatment with (a) proinflammatory agents and (b) proinflammatory agents and 2.5 mM IP6 for 3 h and 12 h. The results are presented as mean ± SD of three separate experiments; *P < 0.05 versus control Caco-2 cells; ^#^ *P* < 0.05 versus proinflammatory agents-stimulated cells.

Over the period of the experiment, a statistically significant decrease in the expression of T*β*RII in cultures treated with LPS of *E. coli* in relation to control (*P* < 0.05) was detected. By comparison, IP6 stimulated LPS-decreased transcription of T*β*RII for both 3 h (*P* = 0.017) and 12 h (*P* = 0.003) (Figures 5(a) and 5(b)). The transcription of T*β*RII did not differ in the control cells and the cells stimulated with LPS of *Salmonella* for 3 h (*P* = 0.129). Moreover, cultures treated with LPS and LPS/IP6 revealed similar level of T*β*RII transcript at this time point (*P* = 0.468). Cell culturing with *Salmonella tyhpi* LPS for 12 h decreased T*β*RII mRNA level as compared to the control (*P* < 0.001). Transcriptional activity of this gene was upregulated in response to 2.5 mM IP6 (*P* = 0.001) (Figures 5(a) and 5(b)). Exposure of Caco-2 to IL-1*β* for both 3 h and 12 h resulted in above 3-fold up-regulation of T*β*RII gene as compared with untreated cells (*P* < 0.05). At 3 h, T*β*RII gene was found to be expressed at the same level in IL-1*β*-stimulated cells and cells treated with both IL-1*β* and IP6 (*P* = 0.312). In longer-lasting cultures, transcriptional activity of this gene was significantly suppressed by IP6 in cells treated with IL-1*β*/IP6 in comparison to those challenged with IL-1*β* alone (*P* = 0.023) (Figures 5(a) and 5(b)).

### 3.6. The Effect of IP6 on T*β*RIII Expression in Caco-2 Cells Stimulated by Bacterial Endotoxins and IL-1*β*

**Figure 6:**
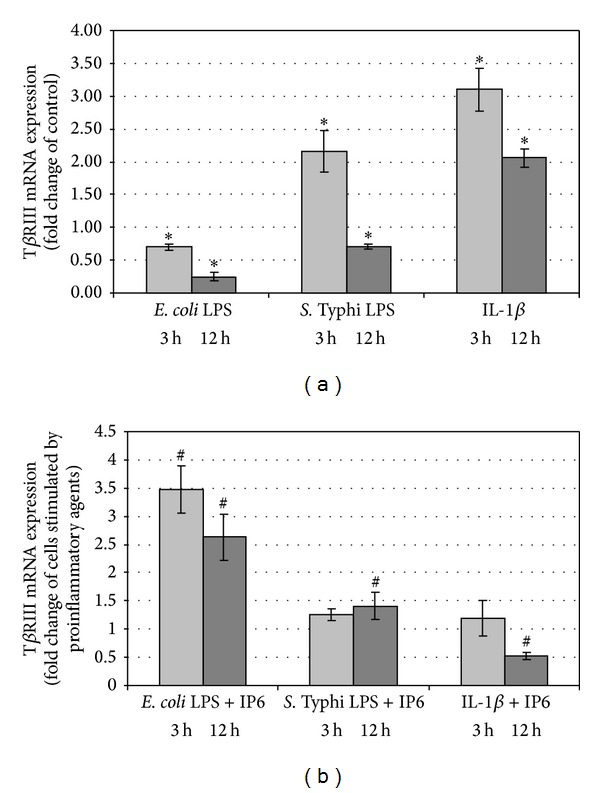
Expression of T*β*RIII gene in Caco-2 cells as determined by real-time RT-PCR. Changes in T*β*RIII mRNA expression in Caco-2 cells after treatment with (a) proinflammatory agents and (b) proinflammatory agents and 2.5 mM IP6 for 3 h and 12 h. The results are presented as mean ± SD of three separate experiments; *P < 0.05 versus control Caco-2 cells; ^#^ *P* < 0.05 versus proinflammatory agents-stimulated cells.

In 3 h and 12 h lasting cultures, the expression of T*β*RIII was significantly lowered by LPS of *E. coli* compared to control cells (*P* < 0.05) (Figure 6(a)). LPS-treated cells exposed to IP6 presented an increase in transcriptional activity of the gene in comparison to cells incubated with LPS only (*P* < 0.001). The considerable, that is, 3.5-fold and 2.6-fold enhancement of T*β*RIII mRNA expression was observed after 3 h and 12 h, respectively, (*P* < 0.05) (Figures 6(a) and 6(b)). Cells exposed to LPS of *S*. Typhi for 3 h produced significantly higher quantity of T*β*RIII transcript (*P* < 0.001) which has not been changed by IP6 (*P* > 0.05). Furthermore, after 12 h, endotoxin of *S*. Typhi reduced transcription of the gene encoding type III receptor (*P* = 0.043), which was markedly up-regulated by IP6 (*P* = 0.041). Treatment of cells with IL-1*β* for both 3 h and 12 h showed a significant (*P* < 0.0001) increase in the expression of T*β*RIII in comparison with control. However, no statistically significant change in its mRNA level in cultures with IL-*β*/IP6 and IL-1*β* only was detected after 3 h (*P* = 0.130). After 12 h, a marked decrease in IL-1*β*-enhanced T*β*RIII transcript level was observed in response to IP6 (*P* < 0.001) (Figures 6(a) and 6(b)).

## 4. Discussion and Conclusions

The researches over the past few years have shown unique and essential roles for TGF-*β* in regulating inflammatory and adaptive immune responses. In particular, TGF-*β* antagonizes the activation of the key proinflammatory cytokines including IL-1*β* and TNF-*α* [13, 32].

The anti-inflammatory treatment strategies can rely on inhibition of proinflammatory cytokine production, receptor binding, signaling, or induction/up-regulation of anti-inflammatory and immunoregulatory cytokines [2]. Various synthetic and natural compounds showing anti-inflammatory properties have been identified. Studies conducted *in vitro* and *in vivo* have demonstrated that phytochemicals, such as curcumin, resveratrol, and genistein, exert chemopreventive effect by targeting the constituents of inflammatory signal pathways [32, 33].

IP6, a natural phytochemical, has recently been described as having immunoregulatory properties. The studies of Cherng et al. [34] showed that IP6 significantly suppressed the secretion of IL-10 and augmented IFN-*γ* production in human peripheral blood mononuclear cells. This compound can modulate the inflammatory response of IEC by regulating their expression and secretion of cytokines and chemokines. Our previous studies revealed that IP6 down-regulated both the IL-1*β*-stimulated increase of IL-8 release from enterocytes and the cellular response to bacterial LPS [35]. Moreover, it appeared to influence the expression of TNF-*α*, proinflammatory cytokine, and its receptors TNFRI and TNFRII in colon cancer cells [36]. IP6 could also inhibit IL-1*β*-stimulated expression of IL-6 and IL-8 at the transcriptional level in IEC [37].

In the present study, we evaluated the influence of IP6 on the expression of TGF-*β*1, -*β*2, and -*β*3 and their receptors T*β*RI, T*β*RII, and T*β*RIII in human intestinal cells under inflammatory conditions. The intestinal epithelium plays important roles in maintaining immune homeostasis in the gut and participates in maintenance of tolerance toward the microflora and food antigens [38]. The cells of intestinal epithelium are capable of producing and releasing IL-1*β*, IL-6, TNF-*α*, and TGF-*β*, either spontaneously or during the course of intestinal mucosa inflammation [35, 38–40]. Therefore, we used LPS derived from *E. coli* and *S*. Typhi, as well as IL-1*β* as a relevant *in vitro* model, to study the regulation of TGF-*β* isoforms and their receptors expression in colon epithelial cells. The cell line Caco-2 (enterocyte-like) utilized in the present experiment is a well-established and widely used model of human intestinal barrier [41].

Lipopolysaccharide released from Gram-negative bacteria cell surface is one of the most potent innate immune-activating stimuli known [42]. Over the last years, the effects of enteropathogenic and enteroinvasive bacteria and members of the normal intestinal microflora on the expression of TGF-*β*s were examined. However, these studies revealed that commensal and pathogenic species induced fundamentally different cytokine responses in human intestinal epithelial cell lines [43]. The results of Zeuthen et al. [38] showed that the presence and composition of enteric bacteria affects the production of IEC-derived TGF-*β* and that the modulatory effect of this cytokine is highly dependent on the bacterial stimulus. The authors indicated relatively high production of TGF-*β*1 in nonstimulated Caco-2 cells and its further increase by stimulation with both G-positive and G-negative commensals [38]. The results of Yoshioka et al. [40] studies demonstrated that intestinal epithelial DLD1 cells increased TGF-*β*1 and LoVo cells increased TGF-*β*2 secretion, at 12 h in response to *E. coli* LPS. However, no significant changes in TGF-*β*s production in hepatocellular carcinoma and myelomonocytic cell lines were observed following stimulation with LPS. Considerable upexpression of mRNAs for TGF-*β*1, TGF-*β*2, and TGF-*β*3 was detected in human intestinal line HT-29 after 3 h coculture with enterotoxigenic *E. coli.* Furthermore, the infection the cells with both enteropathogenic *E. coli* and *S. typhimurium* led to the high induction of TGF-*β*3 mRNA only. There were insignificant differences in TGF-*β*1 and TGF-*β*2 mRNAs in control and cells exposed to both pathogens. Additionally, the authors indicated significantly reduced expression of all TGF-*β* isoforms in HT-29 incubated with commensal bacteria. The majority of bacteria also reduced TGF-*β*1 expression in Caco-2 cell line. Also, a significant increase in TGF-*β*2 and decrease in TGF-*β*3 mRNAs in these cells co-cultured with commensal *E. coli* were determined [43].

In our study, LPS derived from *E. coli* down-regulated mRNA levels of TGF-*β*2 and TGF-*β*3 and all types of receptors in Caco-2 cells over the course of the experiment. In the case of TGF-*β*1, its reduced transcriptional activity was seen after 3 h of incubation. However, a longer stimulation of cells with LPS caused up-expression of this isoform. IP6 elicited the opposed to *E. coli* LPS effect by increasing downregulated transcription of the examined genes and by suppressing the expression of TGF-*β*1 at 12 h. Then, endotoxin of *S*. Typhi promoted differential expression profile of TGF-*β*s and their receptors in a time-dependent manner. The addition of *S*. Typhi. LPS to the cell cultures was manifested by higher transcriptional activity of TGF-*β*2, -*β*3, T*β*RI, and T*β*RII at 3 h, but longer stimulation of Caco-2 resulted in up-regulation of TGF-*β*1 and all receptors. IP6 had no effect on LPS-altered expression of TGF-*β*1. However, it enhanced LPS-increased mRNA expression of TGF-*β*2 and -*β*3. Likewise, this agent was capable of activating LPS-downregulated transcription of T*β*RI, II and III after 12 h.

According to the published data, the proinflammatory cytokines like IL-1, TNF-*α*, and IFN-*γ* increased the production of TGF-*β* isoforms [44]. The main source of IL-1 in IBD patients is the monocyte/macrophage system and active IL-1*β* is released into the colonic mucosa [45]. Low concentrations of IL-1*β* have been shown to induce local inflammatory response followed by the activation of protective immune response [46].

As shown in this study, IL-1*β* stimulation of the epithelial cells up-regulated mRNA levels of all TGF-*β* isoforms and their receptors at both 3 h and 12 h. IP6 counteracted the stimulatory effect of IL-1*β* on TGF-*β*1, T*β*RII, and T*β*RIII genes expression in Caco-2 cells by decreasing their mRNA levels in 12 h lasting cultures. Furthermore, IP6 acted synergistically with IL-1*β* by enhancing the transcription of TGF-*β*2 and -*β*3 isoforms at 3 h.

TGB-*β*s exert their effects via activation of heteromeric receptor complexes of T*β*RI and T*β*RII. They can also interact with the type III receptor. This receptor acts as an enhancer of TGF-*β*s activities by promoting their access to the signaling receptors, especially that of TGF-*β*2 isoform which has a low affinity for the type II receptor [47]. The differential impact of IP6 on the expression of mRNA TGF-*β*s and their receptors in colon epithelium under inflammatory conditions may be related to the role which these isoforms play. The three isoforms of TGF-*β* are distributed in specific spatial and temporal patterns in the tissues and demonstrate distinct biological activities. The TGF-*β*2 and -*β*3 isoforms, whose expression was enhanced by IP6, are the effective inhibitors of epithelial proliferation. TGF-*β*3 may be most effective in inducing epithelial wound repair [48]. TGF-*β*2 suppresses IFN-*γ* and IL-1 at the transcriptional level and plays a critical role in the development of tolerance and the prevention of autoimmunity and anti-inflammatory responses [49]. Also, the increased expression of TGF-*β*1 mRNA in Caco-2 cells treated with IP6 for 3 h following *E. coli* endotoxin pretreatment may indicate its anti-inflammatory activity in relation to this LPS. A variety of pathogenic and proinflammatory stimuli upregulate SMAD7 mRNA expression, which in turn suppresses the TGF-*β* pathway, through activation of NF-*κ*B. Bitzer et al. [13] show that p65/RelA subunit of NF-*κ*B is required for transcriptional activation of SMAD7 by bacterial LPS and the proinflammatory cytokines (IL-1*β*, TNF-*α*). Inositol hexaphosphate exerts influence on cells via phosphatidylinositol-3 kinase (PI3K), MAPK, PKC, AP-1, and NF-*κ*B [28, 29]. Our previously published data demonstrated that IP6 modulated the expression of p65 subunit of nuclear factor *κ*B and its I*κ*B*α* inhibitor in the intestinal epithelial cells [31].

Diseases characterized by chronic inflammation frequently result in irreversible organ dysfunction due to extensive tissue fibrosis. Intestinal fibrosis is often a part of the natural course of IBD. TGF-*β*, in particular the TGF-*β*1 isoform, is a potent profibrogenic agent inducing collagen synthesis and regulating the balance between matrix-degrading metalloproteinases (MMPs) and their inhibitors (TIMPs) [50]. According to Hong et al. [5], natural or synthesized agents that suppress and blockade TGF-*β* signaling generally demonstrate anti-inflammatory and anti-fibrotic activities. Rahal et al. [51] underline that, there are no IBD therapies that have been shown to specifically decrease fibrosis. They investigated the ability of resveratrol, a naturally occurring phytochemical, to decrease inflammation and fibrosis in an animal model of CD and showed the reduction of inflammatory cytokines as a promising trend in decreasing tissue fibrosis. The results of the present study demonstrate that IP6 is able to significantly downregulate TGF-*β*1, T*β*RII, and T*β*RIII activities in colon epithelial cells stimulated with proinflammatory agents IL-1*β* and *E. coli* LPS. Moreover, in the recently published studies, we reported that IP6 influenced constitutive expression of both MMP and TIMP genes and downregulated IL-1*β*-stimulated transcription of some of these genes in the intestinal epithelial cells [52]. Taken together, we postulate that IP6 can attenuate inflammation and fibrosis in intestinal epithelium. Our results were consistent with the report of Kamp et al., [53] who concluded that IP6 reduced pulmonary inflammation and fibrosis in the respiratory bronchioles of rats.

In summary, the present findings suggest that IP6 can suppress the inflammation and exert chemopreventive activity through the modulation of expression of genes encoding TGF-*β*s and their receptors. The current data confirm our previous conclusions that IP6 present in the intestinal milieu may exert immunoregulatory effects on colonic epithelium under inflammatory conditions or during microbe-induced infection/inflammation in order to maintain the colonic mucosa in a noninflammatory state or to counteract infection [35, 37]. Inositol hexaphosphate with its anti-inflammatory and antifibrotic properties seems to be an ideal drug candidate to adjunct therapy of IBD and inflammation-associated colon cancer. Therefore, it is tempting to hypothesize that supplementing the diet of IP6 could be beneficial for preventing or reducing the inflammatory reactions and fibrosis in the intestine.

## Figures and Tables

**Table 1:** Characteristics of primers used in experiment.

Gene	Primer sequence	Product amplified (bp)	TM (°C)
TGF-*β*1	F: 5′-TGAACCGGCCTTTCCTGCTTCTCATG-3′	151	85
	R: 5′-GCGGAAGTCAATGTACAGCTGCCGC-3′		

TGF-*β*2	F: 5′-TACTACGCCAAGGAGGTTTACAAA-3′	201	80
	R: 5′-TTGTTCAGGCACTCTGGCTTT-3′		

TGF-*β*3	F: 5′-CTGGATTGTGGTTCCATGCA-3′	121	81
	R: 5′-TCCCCGAATGCCTCACAT-3′		

T*β*RI	F: 5′-ACTGGCAGCTGTCATTGCTGGACCAG-3′	201	81
	R: 5′-CTGAGCCAGAACCTGACGTTGTCATATCA-3′		

T*β*RII	F: 5′-GGCTCAACCACCAGGGCATCCAGAT-3′	139	84
	R: 5′-CTCCCCGAGAGCCTGTCCAGATGCT-3′		

T*β*RIII	F: 5′-ACCGTGATGGGCATTGCGTTTGCA-3′	173	85
	R: 5′-GTGCTCTGCGTGCTGCCGATGCTGT-3′		

bp: base pair; TM: temperature of melting.
